# Evaluating the Influences of Health Expenditure, Energy Consumption, and Environmental Pollution on Life Expectancy in Asia

**DOI:** 10.3390/ijerph20054000

**Published:** 2023-02-23

**Authors:** Jan Polcyn, Liton Chandra Voumik, Mohammad Ridwan, Samrat Ray, Viktoriia Vovk

**Affiliations:** 1 Department of Marketing, Sumy State University, 40007 Sumy, Ukraine; 2 Department of Economics, Noakhali Science and Technology University, Noakhali 3814, Bangladesh; 3 Sai Balaji Educational Society, IIMS Pune, Pune 411033, India; 4 Department of Economics, Stanislaw Staszic State University of Applied Sciences in Pila, 64-920 Pila, Poland

**Keywords:** Asia, CS-ARDL, energy consumption, economic growth, health outcomes

## Abstract

This study examines the effects of health expenditure, energy consumption, CO_2_ emissions, population size, and income on health outcomes in 46 Asian nations between 1997 and 2019. Cross-sectional dependence (CSD) and slope heterogeneity (SH) tests are utilized due to the close linkages between Asian nations as a result of commerce, tourism, religion, and international agreements. The research uses unit root and cointegration tests of the second generation after validating CSD and SH issues. Due to the results of the CSD and SH tests, it is clear that conventional methods of estimation are inappropriate, so a new panel method, the inter autoregressive distributive lag (CS-ARDL) model, is used instead. In addition to CS-ARDL, the study’s results were checked with a common correlated effects mean group (CCEMG) method and an augmented mean group (AMG) method. According to the CS-ARDL study, higher rates of energy use and healthcare spending lead to better health outcomes for Asian countries over the long run. CO_2_ emissions are shown to be harmful to human health, according to the study. The influence of a population’s size on health outcomes is shown to be negative in the CS-ARDL and CCEMG, but favorable in the AMG. Only the AMG coefficient is significant. In most instances, the results of the AMG and CCEMG corroborate the results of the CS-ARDL. Among all the factors influencing life expectancy in Asian countries, healthcare spending is the most influential. Hence, to improve health outcomes, Asian countries need to take the required actions to boost health spending, energy consumption, and long-term economic growth. To achieve the best possible health outcomes, Asian countries should also reduce their CO_2_ emissions.

## 1. Introduction

Health and economics are unrelated concepts. However, this does not imply that the two most crucial components of a nation’s existence cannot be simultaneously improved. If a country is concerned about maximizing its economy, it must prioritize improving its inhabitants’ healthcare standards. One of the most important things a nation can do for its citizens and its growth on the world stage is to improve its health. A nation’s economic development may be maximized by prioritizing healthcare.

Although there is no unique health index to measure health outcomes, many indicators are used to measure health outcomes [1]. Kindig and Stoddart [2] indicated expected lifespan and the quality of wellbeing as indicators of health outcomes, whereas Erickson et al. [3] emphasized the years of a healthy life. The Canadian National Population Health Survey uses the Health Utilities Index to measure health outcomes [4]. Or [1] used gender-specific potential years of life lost (PYLL) as an indicator, meanwhile also including the mortality rate and the infant or premature mortality rate as some indicators of health outcomes. Well [5] noted that some measurements of health outcomes are life expectancy at birth, anemia, low birth weight, etc. Health outcomes are a key component of human resources; they are crucial in measuring economic progress [6]. Similarly, health outcomes depend on the income of a people and their surrounding environment, along with other factors [7]. Consequently, health outcomes, economic growth, and energy consumption are organically related. Better health outcomes not only induce population expansion; they also boost the working abilities of the labor force, thus accelerating economic growth. Again, population expansion will require more energy to sustain the population’s growth pace. In contrast to economic development, industry flourishes, resulting in many environmental pollutants such as CO_2_ emissions, waste, etc. [8].

**Figure 1:**
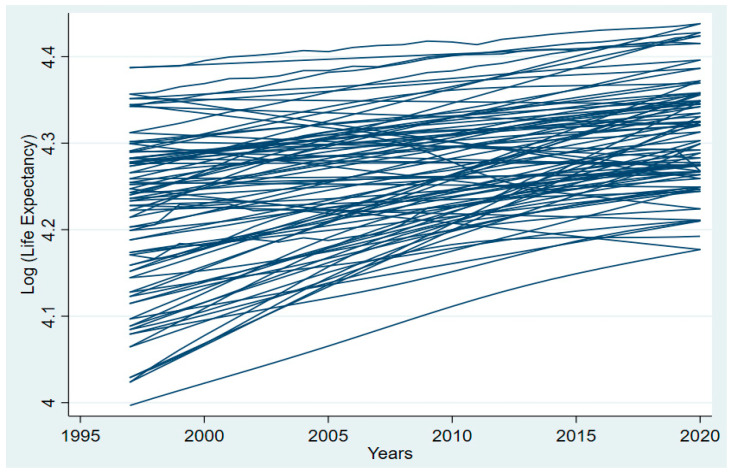
Log of life expectancy over the years in Asia.

Figure 1 shows that increases in both longevity and health have contributed to a dramatic increase in life expectancy in recent decades across Asia. In 1997, health outcomes were 4.2, and in 2019, the log of health outcomes improved to 4.3 in Asia. Improved healthcare infrastructure, wider availability of medical technologies and pharmaceuticals, and progress in medical research are all contributing factors to these outcomes. Higher incomes have led to better nutrition and living circumstances, as well as expanded access to energy consumption, all of which have contributed significantly to economic growth and development. Social progress has helped to increase life expectancy by encouraging people to lead healthier lives, decreasing their use of cigarettes and alcohol, and enhancing public health policy. If the trend of rising life expectancy in Asia continues, it will be a welcome sign of the region’s prosperity and future potential.

The 46 nations and territories of the Asia Pacific Region of the World Health Organization (WHO) cover a huge, densely inhabited land region and an equally enormous, sparsely populated ocean. The area encompasses 21% of the earth’s surface and is home to 53% of the global population. The list of Asian nations where the study was conducted can be found in Appendix A. Even though the region has had impressive economic growth in recent years, the advantages of such growth are not equally dispersed, with several nations and a worryingly sizable portion of the people being left behind in the development process, living in poverty and deteriorating health [9]. The Asia Pacific region has more impoverished people than the rest of the globe combined [10]. Although the area has some significant historical and cultural linkages, it is not uniform. The huge differences between and within Asia Pacific nations present a major public health problem. Annually, hundreds of millions of people are at risk from epidemics and natural catastrophes, and in some fast-emerging countries, health disparities are widening. Environmental, economic, and metabolic illnesses have accompanied economic expansion [11]. Consequently, it is vital to assess the state of health of the Asian continent and to determine the variables that influence health outcomes.

Health and economic growth are important in Asian nations considering their geographic location [12]. In terms of both nominal GDP and PPP, Asia has the world’s biggest regional economy [13]. According to figures provided using IMF [14] data, the nominal size of the 46-nation Asian economy in 2021 was estimated to be approximately USD 36.8 trillion. Asia is responsible for 39% of the global GDP. Its shares are over 47.5% of the global USD 68.7 trillion in PPP terms. After surpassing Europe in 2010, Asia has had the biggest regional economy [13]. In nominal terms, Asia outranks North America, which is placed second by USD 10 trillion. Asia’s GDP is twice as large as Europe’s, ranked second in PPP terms. Whereas the economies of a few Asian nations have expanded rapidly in recent years, most of the continent’s inhabitants have not shared in these benefits [15]. According to the World Bank [16], 36% of South Asians and 14% of East Asian and Pacific residents earn less than USD 1.25 a day. Historically, three main factors—better nutrition, improved infrastructure for public health (such as improved sanitation and the quality of freshwater resources), and enhanced medical technology—have all been linked to economic development in the long term [17]. Numerous research findings demonstrate this well-known connection between income and health within and across countries [18,19]. People live longer and endure fewer years of impairment while living in countries with higher average wages [20]. Anemia is less prevalent among women in countries with higher incomes. The birth weights of infants in these nations are higher. People with greater earnings have a longer lifespan within nations. Children from wealthy backgrounds tend to have better health. Infant and child mortality rates, in addition to child disease, are decreasing [17]. Even if economic development and health have a substantial relationship, there is very little research in Asia that is related to this issue. As such, it is important to figure out how economic growth impacts health outcomes in Asia.

Together, the Asia Pacific region makes up a sizable, varied, and energetic area that is home to 4.7 billion people who reside in nations that span from the largest energy consumer to island countries that are the most susceptible to climate change’s consequences [21]. The area consumes more than half of the energy used globally, with fossil fuels making up 85% of total consumption [16]. Over the previous ten years, electrification rates have increased significantly, reaching 96.6 percent in the region in 2019 [21]. Alternative and renewable energy’s percentage in global final energy consumption is growing quickly, achieving more than 8.5% in 2018 [22]. The greatest improvements were seen in power generation, where renewable energy made up 22.1% of all electricity produced in 2018, up gradually from 16.1% in 2010 [21]. Creating power and heat via the combustion of fossil fuels—gas, coal, or oil—results in significant emissions of GHG, such as CO_2_ and NO_2_ [23]. Numerous adverse consequences on health have been attributed to prolonged exposure to CO_2_. Low cognitive function, difficulty making decisions, fatigue, nausea, vomiting, and tingling feelings are among the symptoms that may arise [24]. Thus, it is important to analyze how energy consumption is related to health outcomes in the Asia region.

In this work, we have concentrated on finding the linkage between economic progress, energy usage, and health in selected Asian countries. The objectives of this study are to determine (i) the effect of economic growth on health outcomes; (ii) the effect of energy usage on health outcomes; and (iii) whether CO_2_ emissions have a meaningful impact on health outcomes.

Not only does energy consumption increase economic activity, but it also increases CO_2_ emissions. Even though the impact of energy growth on health outcomes in this region has been identified, many previous studies still need to pay attention to the linkage between energy usage, economic development, and health outcomes. Specifically in Asian nations, there are no recognized works on this topic. Evidentially, a healthy population is positively and significantly associated with economic expansion. In their seminal research, Bloom et al. [25] looked at how people’s health impacts their country’s GDP. Rarely has the causality between economic growth and health outcomes been examined. Furthermore, the linkage between energy usage and health effects still needs to be proven. Evaluating the impacts of energy usage and economic expansion on health outcomes in Asian countries is consequently of utmost importance. The subsequent sections of this paper provide a literature review and statistical analysis relating to the relevant data, and the remaining sections discuss methodology, the empirical findings, and conclusions.

The countries of Asia are competing to become the world’s economic leaders. Economic output, population, healthcare spending, and energy use are all rising in these nations [26]. Therefore, it is crucial to assess the effect of these variables on health outcomes. In light of these presumptions, this paper makes the following contributions to the current literature. The first part of the study looks at how population, income, health expenditure, and energy consumption have affected life expectancy in Asia between 1997 and 2019. Second, there is a great deal of interaction between Asian countries due to trade, religion, bilateral cooperation, and cultural exchange. There is evidence of CSD and SH issues in the data. Therefore, the paper uses cointegration tests, a cross-sectional unit root test, and CS-ARDL of the second generation to deal with data issues. To check the robustness the paper, we applied AMG and CCEMG estimators. The theoretical framework of this research is an application of Smith’s [27] health production model to determine the effect on health outcomes. Finally, the study provides recommendations for how policymakers may approach policymaking in light issues related to income, CO_2_ emissions, population, and energy usage.

## 2. Literature Review

Several investigations have been undertaken on the links between health, economic growth, and energy usage. These studies proceed country by country or worldwide.

Mankiw et al. [28] used health as a human resource factor in their economic development analysis. Barro [6] presented a growth model that used health capital, physical capital inputs, hours worked, and education level. Therefore, based on Mankiw et al. [28], health capital is considered to be a regular production indicator. Lucas [29] discovered a similar finding. Energy use and economic growth have been studied extensively. Granger’s causality and mediation model was employed by Gyimah et al. [30] to explore the connection between the utilization of sustainable energy and economic expansion for the scenario of Ghana. The research findings indicated that the use of sustainable energy sources can have a sizeable and beneficial influence on economic growth [31]. Tutak and Brodny [32] discovered that using energy from sustainable energy sources has a favorable impact on economic development, lowering GHG emissions and minimizing traditional energy usage in almost all European Union nations [33]. Renewable energy consumption (REC) and economic growth (EG) have a bidirectional, unidirectional, or no causal link, according to empirical studies. Ocal and Aslan [34] studied EG and REC in Turkey. Using data from 1990 to 2010, they found that EG causes REC. Sadorsky [35] and Salim et al. [36] found unidirectional causation from EG to REC for 18 developing nations, Romania, and OECD countries. Numerous studies, including those of Apergis and Payne [37], Ito [38], Magazzino [39], Fotourehchi [40], and Khobai and Le Roux [41], have observed a unidirectional causality from REC to EG.

Mujtabe and Sahazad [42] conducted a study in OECD nations and discovered that a causality exists in the long term between renewable energy and healthcare spending. Majeed et al. [43] examined the connection between renewable energy consumption and health outcomes in 155 economies using panel approaches such as two-stage least squares, random effects, fixed effects, pooled OLS, and the generalized method of moment (GMM). It has been empirically demonstrated that using renewable energy is beneficial to health. Using sustainable energy has been shown to prolong the average life span and decrease fatality rates. The favorable association between sustainable energy and public health indicates that sustainable energy assists in managing chronic illnesses, increases life expectancy, and reduces mortality and tuberculosis rates. Using the GMM methodology, Hanif [44] explored the association between different patterns of energy use and the state of human health in Sub-Saharan Africa. The use of solid fuels (wood pellets, peat, charcoal, wood, agricultural waste) for cooking and the consumption of fossil fuels (gas, coal, oil) is dramatically raising the incidence of TB, according to the study’s findings. Moreover, the data demonstrate that the usage of both fossil and solid fuels has negative impacts on the expected life span in Sub-Saharan African nations by increasing the death rate. The findings suggest that economic growth and the use of renewable energy sources such as wind, sun, and water (which can prevent residents from overexposure to particulate matter and toxic pollutants) contribute to reducing mortality and managing tuberculosis.

Using time series data from Turkey, Essen and Çelik Keçili [45] assessed the ways in which the development of an economy and health spending are related. The study found a substantial positive linkage between economic expansion and healthcare spending, employing the Granger causality and Johansen cointegration test. Chen et al. [46] researched the ways in which factors related to economic development and the environment influence life expectancy in 20 different emerging and developed economies. The correlation coefficients between the variables were evaluated using the Pearson correlation coefficient, and the influence of each indicator on LE was determined using multiple regression models. The research found that the per capita income had a considerable favorable effect on life span in developed and developing nations. Using a full information maximum likelihood model, Miladinov [47] explored the linkage between GDP growth and life expectancy at birth in the five EU membership candidate nations (Albania, Bosnia and Macedonia, Montenegro and Herzegovina, and Serbia). The research results demonstrated that longer life expectancy is associated with greater levels of income and lower neonatal mortality rates. Applying time series data from Pakistan, Wang et al. [48] researched the correlation between life expectancy and economic expansion. Utilizing the ARDL bound testing method, the research found that economic growth is positively related to life expectancy.

Youssef et al. [49] used the SUR technique to determine the causal linkage between energy usage and health outcomes. The study’s results showed that health and energy consumption have a healthy relationship. Wang [50] examined the influence of energy usage on public health and the environment using the exposure-response approach. The findings exhibited that energy use impacts both beneficial and harmful health considerations. Arawomo et al. [51] evaluated the dynamic connection between economic development, energy use, and health in Sub-Saharan Africa. The results of the analysis exhibited that neither energy use nor economic growth has a substantial effect on health outcomes. Smith et al. [52] showed a unilateral causation for the association between energy use and health outcomes, but Youssef et al. [49] found a bidirectional causality. Smith et al. [52] found a positive and inverse linkage between energy use and health outcomes. As such, it is evident that the association between health and economic growth has been thoroughly examined under the structure of growth theories, as shown in the above literature review.

Our assessment of the literature reveals that no research has examined how energy consumption, health expenditure, and income affect health outcomes for a panel of Asian countries, and those studies on the linkage between energy use and health outcomes are scant and inconsistent, often ignoring potential CSD and SH in panel data, leading to erroneous estimations. Previous research ignored CSD and SH problems. It is important to fill this massive gap in the literature. Because of the lack of data, the conflicting results, and the methodological flaws in the existing studies, the authors opted to investigate the connection between energy use, GDP growth, and health outcomes in a sample of Asian nations.

## 3. Methodology

### 3.1. Theoretical Framework and Model Specification

Smith [27] suggests the following outline for the health production function:

(1) $$\mathrm{HO}\;=\;f\;\left(\;M.E\;\right)$$

The health production function developed by Smith [27] that is shown in Equation (1) postulates the relationship between medical and non-medical input combinations and the resulting output. As a result, health production relies on non-medical, socioeconomic, financial, and physical elements in addition to the healthcare system and its resource input. Arawomo et al. [51] used the health production function in its general version proposed by Smith [27] to examine the dynamic link between economic development, energy consumption, and life expectancy at birth in SSA economies. The letters “HO” represents health outcomes, “M” stands for medical resources, and “E” refers to non-medical, social, economic, and lifestyle factors. According to the hypothesis, health outcomes will improve in tandem with the growth of healthcare spending (M). Consequently, expanding medical resources may achieve better healthcare for the populace. However, there is another case in which the law of diminishing returns comes into play.

Health outcomes can also be measured by social, economic, and physical aspects [6]. The health production function proposed by Or (2000) serves as the theoretical model for this investigation. Or [1] divides non-medical factors into three categories: physical environment, lifestyle, and socioeconomic factors. According to Or [1], all the factors that individuals can control, such as liquor consumption, food habits, working out, and individual hygiene, significantly impact health.

According to Or [1], the specific model for the study is:

(2) $${\mathrm{HO}}_{t}={\;\alpha }_{0}+{\;\alpha }_{1}M_{t}+{\;\alpha }_{2}N_{t}+{€}_{t}$$

In Equation (2), to simplify, let us say that HO is the health outcome (as measured by life expectancy), M is a vector of a medical variable (as measured by healthcare spending), and N is a vector of non-medical variables (as measured by energy consumption, income, and education) [53]. Here, $\alpha_{0}$ represents the intercept coefficient, and $\alpha_{1}$ and $\alpha_{2}$ represent partial slope coefficients. The $\alpha_{0}$ remains constant throughout the period. ${€}_{t}$ represents the error term in the aforementioned equation.

Now, we can relate health status with various factors of interest. The more specific form of the above health equation is given in Equation (3):

(3) $${\mathrm{HO}}_{t}=\beta_{0}+\beta_{1}\mathrm{EC}+\beta_{2}\mathrm{GDPpc}+\beta_{3}\mathrm{HEX}+\beta_{4}\mathrm{POP}+\beta_{5}{\mathrm{CO}}_{2}+{€}_{t}$$

Here, HOt is the health outcome, EC is used for energy consumption, GDPpc indicates GDP per capita, HEX shows general health expenditure, POP is used for population, and CO_2_ denotes CO_2_ emissions. If the slope coefficients of the variables become greater than zero or positive (β > 0), this means that the independent variable is positively related to the dependent variable when all other explanatory variables remain constant. In Equation (4), the variables were transformed into logarithmic form. The logarithmic transformation can help to normalize the distribution, making it more symmetrical and more similar to a normal distribution. Many statistical tests assume that the data are normally distributed, so this can be useful. By transforming a skewed variable into one with a more normal distribution, statistical tests can yield more precise and meaningful results. In addition, logarithmic transformations can be used to stabilize the variance of a variable, thereby reducing the impact of extreme values on the analysis.

Therefore, the estimated model is as follows:

(4) $${\mathrm{lnHO}}_{t}=\beta_{0}+\beta_{1}\mathrm{lnEC}+\beta_{2}\mathrm{lnGDPpc}+\beta_{3}\mathrm{lnHEX}+\beta_{4}\mathrm{lnPOP}+\beta_{5}{\mathrm{lnCO}}_{2}+{€}_{t}$$

In Equation (4), all variables used in this analysis are shown in logarithmic form.

### 3.2. Data

Utilizing secondary data, the econometric outcome was estimated. The World Bank produces the WDI, which was employed as a secondary source of information. This research studied life expectancy as an illustration of how public health is influential. GDP per capita indicates the performance of the economy. The paper also considers several other control factors, such as energy usage, population, CO_2_ emissions, and health expenditures, to identify the influencing factors on health outcomes. Table 1 presents a more comprehensive investigation of the information for 46 Asian economies from 1997 to 2019. The dependent variable, life expectancy at birth, measured in years, is used as a proxy for health outcomes in the economies of Asia. All variables were transformed into logarithms for the study. Logarithmic transformation was applied to variables for a number of reasons in this study. Variables with high skewness can be transformed into more normal distributions via logarithmic transformations. A non-linear connection can be made linear by applying a log transformation to one or more of the variables involved. Variances can take on a variety of forms when working with data that span a broad range of values. Data transformation can stabilize variance between groups and lessen variability. With a log transformation, the findings are more understandable and straightforward to relay.

Table 2 presents the descriptive statistics for the variables from 1997 to 2020. Means, medians, standard deviations, extreme values, and ranges are all provided for each series. Natural logarithms of health outcome (lnHO), energy consumption (lnEC), gross domestic product (lnGDP), health spending (lnHEX), population (lnpop), and carbon dioxide emissions (lnCO_2_) are displayed in the table, along with descriptive data for these six variables. For each variable, we present the number of observations, the average, the standard deviation, the lowest and highest values, and the range of values.

Over a thousand observations were made for lnHO, 932 for lnEC, 1014 for lnGDP, 923 for lnHEX, 944 for lnpop, and 958 for lnCO_2_. Variables’ means range from 3.942 for lnEC to 16.46 for lnpop, and their standard deviations range from 0.0806 for lnHO to 2.724 for lnEC. Each variable’s lowest and maximum values are also provided, with lnEC’s range extending from 0 to 6.646 and lnpop’s from 3.997 to 21.07.

### 3.3. Estimation Technique and Econometrics Procedure

These Asian countries may be suffering a stationary CSD, SH, or mixed-order stationary problem, according to the characteristics of the panel data and the cross-sectional connection. The CSD test is used in the paper because of the widespread cooperation and coordination among Asian countries. Even though Asian economies are growing, the rates at which they are expanding vary greatly. This is why the slope homogeneity test is used here. Confirming the CSD and SH issues necessitates using the second-generation unit root test and cointegration test in this effort. As part of CSD and SH management, we use the CIPS [55] tests. The cointegration test follows the unit root test in the paper. Specifically, the paper used a cointegration test of the second generation [56]. The CSD, heterogeneous effects, and nonstationary data issues are all accounted for in the Westerlund [56] test. The CS-ARDL technique was used after all these checks were taken into account in the study. The robustness was tested using AMG and CCEMG estimations, which were also used in the research.

This section may be divided by subheadings. It should provide a concise and precise description of the experimental results, their interpretation, as well as the experimental conclusions that can be drawn.

#### 3.3.1. CSD Test

Because this research utilizes panel data, it is important to examine the data for CSD. The CSD is caused by similar socioeconomic conditions. This test directs the implementation of subsequent tests. Therefore, this study employs Pesaran’s [57] CSD, Frees test, Friedman CSD, and Pesaran abs tests. The CSD equation is shown by the following Equation (5):

(5) $$\mathrm{CSD}=\sqrt{\frac{2T}{N\left(N-1\right)}}(\sum_{j=1}^{m-1}\sum_{i=j+1}^{m}\theta_{\mathrm{ji}}^{t}\;)\;$$

where N represents a cross-sectional dimension, and T represents a time series dimension. $\theta_{\mathrm{ji}}^{t}$ Represents the estimation of the correlation residual.

#### 3.3.2. Slope Homogeneity Test

Equally significant is the conduct of SH. This test examines the data for commonalities. As a result, the Pesaran and Yamagata [58] test is applied. The SH equation is shown below in Equations (6) and (7):

(6) $$\Delta_{\mathrm{SH}\;}^{∼}=N^{\frac{1}{2}}2k^{\frac{1}{2}}\left(\frac{1}{N}S^{∼\;}-k\right)$$

(7) $$\Delta_{\mathrm{ASH}}^{∼}=N^{\frac{1}{2\;}}\left(\frac{2k{\left(T-k-1\right)}^{-}{}^{\frac{1}{2}}}{T+1}\right)\left(\frac{1}{N}S^{∼}-k\right)$$

In Equations (6) and (7), ‘N’ represents a cross-sectional dimension, ‘T’ denotes a time series dimension, and ‘k’ represents the number of explanatory variables. Additionally, $\Delta_{\mathrm{SH}\;}^{∼}$ and $\Delta_{\mathrm{ASH}}^{∼}$ show delta tidle and delta tidle adjusted, accordingly.

#### 3.3.3. Stationarity Test

Additional guidance for identifying the unit root of the data is analyzed by CSD and SH. The level of integration is determined by this test. Following the unit root test, the co-integration test will be conducted. This study used the cross-sectional IPS (CIPS) [55] test. The cross-sectionally augmented IPS (CIPS) unit root test is a statistical method used to test for the presence of a unit root in panel data. A unit root is a feature of a time series in which the series has a stochastic trend, meaning that it can drift away from its mean over time.

The CIPS unit root test is an extension of the IPS test, which is used to test for unit roots in individual time series. The CIPS test is designed to account for the potential presence of cross-sectional dependence in the panel data, which can lead to incorrect inference if not properly addressed.

Equation (8) is utilized for the CIPS test:

(8) $$\mathrm{CIPS}=\frac{1}{N}\sum_{i=1}^{N}t_{i}\left(N,\;T\right)$$

‘N’ represents a cross-sectional dimension, and ‘T’ denotes a time series dimension.

#### 3.3.4. Co-Integration Test

Following the unit root test, the panel cointegration test proposed by Westerlund [56] is used to assess the cointegration. To determine whether two or more non-stationary time series variables are cointegrated, statisticians can apply the Westerlund cointegration test. Cointegration describes the situation in which two or more non-stationary variables exhibit a stable, long-term relationship. Put another way, despite occasional differences, cointegrated variables generally follow the same path across time [59]. This test produces reliable results and incorporates the CD and SH in panel data. The equation for the cointegration test contains the following Equations (9)–(12):

(9) $$G_{t}=\frac{1}{N}\overset{N}{\underset{j=1}{\sum }}\frac{\theta_{j}^{ƭ}}{\mathrm{SE}\theta_{j}^{ƭ}}\;$$

(10) $$G_{a}=\frac{1}{N}\overset{N}{\underset{j=1}{\sum }}\frac{T_{j}^{ƭ}}{\theta_{j}^{ƭ}\left(1\right)}$$

(11) $$P_{t}=\frac{\theta_{j}^{ƭ}}{\mathrm{SE}\left(\theta^{ƭ}\right)}$$

(12) $$\theta^{ƭ}=\frac{P_{a}}{T}$$

$\theta^{ƭ}=\frac{P_{a}}{T}\;$ shows the ratio of correction yearly.

#### 3.3.5. CS-ARDL Methodology

Using the CS-ARDL model, the short- and long-term correlations between energy consumption, economic growth, and health consequences are determined. The CS-ARDL provides a definitive solution, as it is resistant to endogeneity and non-stationarity concerns, and it tackles CSD and heterogeneity issues [60]. Because CSD and slope homogeneity issues exist, the CS-ARDL method applies to this investigation. The following Equation (13) shows the general form of the CS-ARDL model:

(13) $$\Delta {\mathrm{EF}}_{\mathrm{it}}=\theta_{i}+\overset{m}{\underset{j=1}{\sum }}\theta_{\mathrm{it}}\Delta {\mathrm{EF}}_{i,t-j}+\overset{m}{\underset{j=0}{\sum }}\theta_{\mathrm{ij}}^{\prime }+{\mathrm{AEV}}_{i,t-j}+\overset{m}{\underset{j=0}{\sum }}\theta_{\mathrm{it}}^{\prime }{\bar{Z}}_{t-j}+{€}_{i,t}$$

The cross-section average is denoted by ${\bar{Z}}_{t}$, which is equivalent to ($\Delta {\bar{\mathrm{EF}}}_{t},\;{\bar{\mathrm{AEV}}}_{t}\prime )\prime$, where AEV represents all independent variables.

#### 3.3.6. Robustness Check (AMG and CCEMG)

In the case of cross-sectional dependency pitch heterogeneity, the application of conventional methods may provide insufficient estimates. We apply Eberhardt and Bond [61] and Pesaran’s [62] CCEMG in the presence of slope heterogeneity, cross-sectional dependency, and structural breaches. Furthermore, both AMG and CCEMG perform better when estimating using common components that are not stationary and uncertain. The CCEMG takes temporal variations with various pitch factors into account and solves the identification problem. When independent and dependent variables are assessed across all sections rather than just over particular periods or trends, cross-sectional dependency spillover is decreased [63]. The AMG is a particular approach to CCEMG that takes into account yearly incompetence and overlooked factors, as well as cross-dependence, heterogeneity, and structural technical advances.

## 4. Results

Table 3 demonstrates the outcomes of the CSD and indicates the interdependence between the variables. The research applied four CSD tests, so that the findings are robust. In other words, the findings of the Pesaran CSD test, the Frees test, the Friedman test, and the Pesaran abs test are shown, and there is no CSD assumed as the null hypothesis. The results of the CSD test indicate that the null hypothesis should have been rejected at the 1% significance level. This verifies that the dataset contains CSD. Similar social and economic policies account for this cross-sectional dependence.

The results of the slope homogeneity [58] test are shown in Table 4. For the sake of this test, we will assume that slope values are uniform continuously. The findings are shown in Table 4, which reveals that delta tidle is significant at the 5% significance level, and delta tidle adjusted is significant at the 1% significance level. As a consequence, the model is concerned with heterogeneity, and the null hypothesis of homogeneity for slope values is rejected.

Table 5 summarizes the outcomes of the cross-sectionally augmented IPS (CIPS) unit root test. The empirical result of the CIPS unit root test exhibits that lnH, lnEC, lnCO_2_, and lnPOP have unit root problems at the level. After taking the first difference, variables become significant at a 1% significance level, and they are integrated into I(1). The results also depict that lnGDP and lnHEX are significant at a 1% significance level in both the level and first difference. Therefore, the result is that lnGDP and lnHEX are integrated into I (0).

Bootstrap *p*-values are taken into account while analyzing the findings of the ECM test provided by Westerland [56] to determine whether there is a cross-sectional dependence between the series used in the analysis. The findings are interpreted taking into consideration the Group tau (G_t_), Group alpha (G_a_), Panel tau (P_t_), and Panel alpha (P_a_) critical values because of the heterogeneity between the series [64]. The findings of the cointegration test are shown in Table 6. The null hypothesis is that there is no long-term cointegration between the dependent variable and the independent variables. The outcome presented in Table 6 indicates that the null hypothesis should be rejected, because the p values for G_t_ and P_t_ are highly significant at the 1% level of significance. As a consequence, there is long-term cointegration between health outcomes and other independent variables reported in this analysis in the Asian region.

The outcomes of the CS-ARDL are presented in Table 7. The validity of CS-ARDL results has been confirmed by the CCEMG and AMG test results presented in Table 8. The findings of the CS-ARDL results show that energy consumption has a significant positive relationship with health outcomes. The coefficients of lnEC are 0.0019 and 0.00015 in the long and short run, respectively, which means at a 1% level of significance, an increase in energy consumption of 1% will lead to an increase in health outcomes by 0.0019% in the long term and 0.00015% in short term. The results are also validated by the AMG and CCEMG tests. The findings demonstrate that countries in the Asian region consume more eco-friendly resources and renewable energy, and this is why energy consumption has a significant positive impact on health outcomes.

The results reveal a positive relationship between economic growth and health outcomes, but it is insignificant in the long run. Especially the coefficient of lnGDP is positive at 0.0018. Therefore, a 1% increase in GDP per capita will increase health outcomes by 0.0018% in the long run at a significance level of 5%. In the short term, the coefficient of lnGDP exhibits that a 1% increase in GDP will lead to an increase in health outcomes by 0.00062%. CCEMG test results confirm these results. In addition, these results are consistent with the existing research [65,66]. These findings suggest that as the economy grows faster, it will increase the per capita income, and higher-income people can spend more on healthcare. Thus, this study finds a positive correlation between economic growth and health outcomes in the Asian region.

The coefficients of lnHEX are 0.0012 and 0.00034 in the long and short term, respectively, which means that at a 1% significance level, a 1% increase in health expenditures will increase the health outcome by 0.0025% in the long term and 0.00034% in the short term. The test results of AMG and CCEMG confirms the results. The findings suggest that the government’s increase in general health expenditure and growth in health-related services such as hospitals, clinics, and healthcare services will positively impact public health.

The population has no significant relationship with health outcomes in the long and short term. The results were also confirmed by the AMG and CCEMG test results. This means there is no evidence of a significant relationship between health outcomes and population. The CS-ARDL, AMG, and CCEMG results show that CO_2_ emissions have an important negative relationship with health outcomes in the short and long term. The coefficient of lnCO_2_ is −0.0028 in the long term and −0.00119 in the short term and significant at a 1% significance level. The results mean that a 1% increase in CO_2_ will lead to declining health outcomes by 0.0028% in the long term and 0.00119% in the short run. CO_2_ emissions are one of the main reasons for air pollution and adversely affect public health. With the speed adjustment, the coefficient of ECT is −0.5689, indicating that the life expectancy rate converges to its long-run equilibrium by 56.89%.

The robustness results show similarity with our baseline CS-ARDL findings (Table 8). All directions are the same except for the impact of the population in the AMG estimator. The impacts of GDP are positive and significant in both the short and long run in the CS-ARDL model. However, the impact of GDP is insignificant in the AMG estimator. On the other hand, the population impact is insignificant in the CS-ARDL model but significant in AMG estimation.

## 5. Discussion

**Figure 2:**
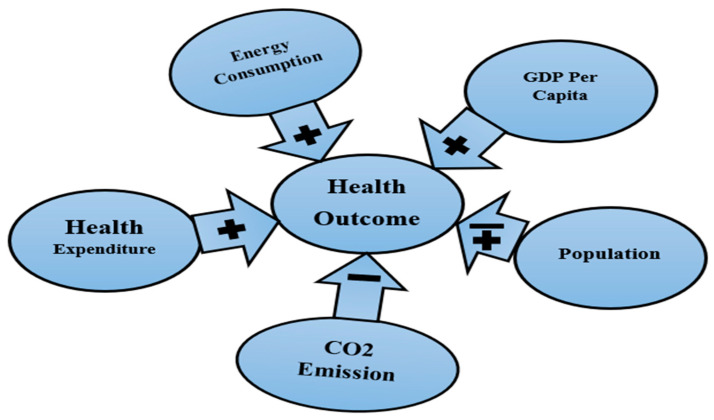
Influencing factors’ signs on health outcome.

Figure 2 graphically shows the influencing factors’ signs on health outcomes. The figure shows that health expenditure, energy consumption, and GDP per capita positively impact life expectancy in all estimators. On the other hand, CO_2_ harms life expectancy in all estimators. Population impacts positively on life expectancy in the AMG estimator and negatively on life expectancy in the CCEMG estimator.

The observations of this study support the notion that overall energy consumption positively influences human health. Energy generated from fossil fuels generates significant CO_2_ emissions and has negative health effects. In contrast, alternative energy sources benefit the environment and human health [46,67,68,69]. Thus, the practical impact of overall energy consumption on health in the Asian area indicates that renewable energy consumption outperforms nonrenewable energy consumption in this region [70]. Renewable energy enhances life expectancy, lowers infant mortality, prevents TB cases by replacing traditional energy sources, and, as a result, enhances environmental quality. In addition, it enhances health outcomes by improving availability, cost, supply, food quality, and nutrition. The study’s findings align with Alharthi et al. [71], Kadria et al. [72], Sasmaz et al. [73], and Majeed et al. [43].

The outcome of this study has found that CO_2_ emissions have a significant negative impact on health outcomes both in the short and long term. Smog, the more obvious type of air pollution, is indirectly caused by carbon dioxide. The production of smog, which is detrimental to respiratory health, is facilitated by the increased warmth and humidity caused by carbon dioxide emissions. The effects of carbon dioxide pollution on the environment and human health are multifaceted and occur in both immediate and delayed ways. These findings are consistent with Emodi et al. [74], Oyedele [75], and Farooq [76].

The findings of this study have also demonstrated that health outcomes are positively influenced by economic growth and health expenditures. A high GDP (gross domestic product) typically corresponds to a large government budget and taxable income. If the government is intelligent, it should allocate a significant portion of the budget to healthcare, cleanliness, and research and development. Countries with excellent management and governance and a high GDP per capita have great food security. This indicates that the people’s food and water are wholesome and do not cause ailments such as food poisoning, diarrhea, etc. More investment in facilities that maintain cleanliness eradicates illnesses such as malaria from the nation. Water and cleanliness indicate that individuals are also healthy. Healthcare is extremely important. It should be cutting-edge, accessible, inexpensive, and continually advancing. Research and development are very important. Modern research and development have enhanced healthcare services. These findings are in line with Hlafa et al. [77], Arthur and Oaikhenan [78], and Oluwatoyin et al. [79].

## 6. Conclusions

This study examines the influences of energy consumption and economic growth on health outcomes in the Asian region from 1997–2020. The effects of carbon dioxide emissions, health expenditures, and the population were also analyzed as control variables that have a crucial impact on health outcomes. The research employs second-generation econometric analytical tests. The study used the slope homogeneity test developed by Pesaran and Yamagata [58] to identify the homogeneity of slope values and the cointegration method established by Westerlund [56] to evaluate the long-run equilibrium link between variables. In this work, the CS-ARDL method is applied to the estimations described by Pesaran and Smith [80] and Chudik and Pesaran [60]. According to Westerlund [56], the ARDL method is required if the panel dimensions are substantially greater than the transversal components (T > N), as is the case in this study. Additionally, we validated the data’s heterogeneity and the presence of CSD. In addition, the CIPS Pesaran [55] unit root analysis revealed a mixed integration order of the examined variables, which applies to the second generation of cointegration techniques. In the long run, all variables except population were cointegrated with health outcomes.

The CS-ARDL results are compatible with the AMG and CCEMG methods’ estimations. This study found that during the period from 1997–2020, energy consumption in selected Asian countries increased health outcomes. The consumption of renewable and eco-friendly resources over non-renewable resources was one of the main reasons for the positive results between total energy consumption and health outcomes. Faster economic growth has also had a significant positive impact on health outcomes. A higher per capita income increases the ability to spend more on healthcare. The findings also revealed a significant negative impact of CO_2_ emissions on health outcomes. Finally, the results of this study found a positive association between health expenditure and health outcomes. The more the government spends on general health services, the higher the quality of public health.

## 7. Policy Recommendation

These findings lead us to propose the following policy changes. There is a direct correlation between the amount of energy consumed and health outcomes. One of the signs of an improved lifestyle is the amount of energy that is consumed. The consumption of renewable energy sources is more favorable than environmental sustainability [81,82]. To reduce greenhouse gas emissions, private sector investment in clean energy is essential. Since our research proves that using fossil fuels harms the environment, and since using renewable energy sources has been shown to enhance the air quality in Asia, we propose that the latter be phased out in favor of the former. The use of renewable energy sources also helps to lower carbon dioxide emissions. Asian countries should prioritize renewable energy sources. The potential for generating renewable energy in Asia, including wind, solar, hydro, and so on, is enormous. The government and policymakers need to pay attention to this. Because of its efficacy in combating poverty, inequality, and income disparities, raising the average level of income is one of the primary goals of economic policy. Increasing GDP in Asia can be achieved without compromising long-term sustainability. A higher standard of living and greater accessibility to healthcare are both confirmed by a higher per capita GDP. The government needs to prioritize GDP per capita and reduce economic disparity. There was also a positive correlation between health outcomes and public and private health spending. Increasing healthcare spending is essential for ensuring a healthy population. The government needs to invest more in healthcare so that residents of outlying areas can reap the benefits. Conversely, CO_2_ emissions have negative effects on human health. Therefore, policymakers should implement many policies to reduce CO_2_ emissions. To discourage carbon dioxide production, Asian nations can implement a carbon tax. Similarly, a larger population was associated with worse health. It is suggested that citizens verify their healthcare institution; awareness should be spread, so that everyone knows the importance of healthcare, the signs of common health problems, and the places in which to find medical help.

## 8. Limitations and Future Research

This study is limited by the availability of data on health expenditure, energy consumption, and environmental pollution, which may vary across different countries and regions in Asia. The study does not consider other factors that influence health, such as genetic factors, lifestyle choices, and socioeconomic factors, which could confound the results. Asia is a large and diverse continent with significant cultural, economic, and environmental variations, which could affect the generalizability of the study findings. Future research could explore the impact of other factors on life expectancy, such as access to healthcare, lifestyle choices, social determinants of health, and climate change. Researchers could use experimental or quasi-experimental designs to establish the causality between health expenditure, energy consumption, environmental pollution, and life expectancy in Asia. Future research could analyze the impact of health expenditure, energy consumption, and environmental pollution on life expectancy at the country level to identify specific policies or interventions that could improve health outcomes. Longitudinal studies could examine the long-term effects of health expenditure, energy consumption, and environmental pollution on life expectancy in Asia.

## Figures and Tables

**Table 1:** List of all variables.

Variable Name	Log	Unit	Source
Health outcomes	lnHO	Total expected life span at birth (years)	World Development Indicator
Energy Consumption	lnEC	Use of energy (kg of oil equivalent per capita)	
GDP per capita	lnGDPpc	GDP per capiata (current USD)	
Health expenditure	lnHEX	Government healthcare spending (in current USD)	
CO_2_ emissions	lnCO_2_	CO_2_ emissions (metric tons per capita)	
Population	lnPOP	Population, total	

Source: WDI (2022) [54].

**Table 2:** Summary statistics.

Variables	Observations	Mean	Std. Dev.	Min	Max
lnHO	1012	4.267	0.0806	3.997	4.438
lnEC	932	3.942	2.724	0	6.646
lnGDP	1014	5.795	1.418	0	6.975
lnHEX	923	4.623	2.482	0	6.782
lnpop	944	16.46	1.907	12.48	21.07
lnCO_2_	958	5.694	1.571	0	6.960

**Table 3:** Results of CSD test.

	H_0_: There Exists a Cross-Sectional Dependence	
	Test Statistics	*p*-Value
Pesaran CSD	56.209 ***	0.000
Frees test	13.566 ***	Alpha = 0.01:0.2034
Friedman test	174.288 ***	0.000
Pesaran abs	56.209 ***	0.001

*** shows 1% significance.

**Table 4:** Slope homogeneity test.

Slope Homogeneity Tests	Δ	*p*-Value
$\overset{ˇ}{\Delta }$ test	31.811 ***	0.033
${\overset{ˇ}{\Delta }}_{adj}$ test	37.797 ***	0.010

The null hypothesis here is that all slope coefficients are homogeneous. *** denotes less than 1% level.

**Table 5:** Panel unit root test.

Variables	Level		First Difference		Order
	without Trend	with Trend	without Trend	with Trend	
Cross-Sectionally Augmented IPS (CIPS)					
lnH	−1.170	−2.197 ***	−3.639 ***	−3.636 ***	I(1)
lnEC	−1.727	−2.809 *	−3.715 ***	−3.706 ***	I(1)
lnGDP	−2.633 **	−2.491 **	−2.066 ***	−2.293 ***	I(0)
lnHEX	−3.837 ***	−3.906 ***	−5.730 ***	−5.900 ***	I(0)
lnPOP	−1.286	−1.774	−4.839 ***	−5.038 ***	I(1)
lnCO_2_	−0.881	−1.438	−4.984 ***	−5.232 ***	I(1)

Note: *, **, and *** explain the level of significance at 10%, 5%, and 1%, respectively, whereas the values in parenthesis contain *p*-values.

**Table 6:** Results of Westerlund test for cointegration.

Variables	Value	Z-Value	*p*-Value
Gt	−2.192	3.235	0.00
Ga	−1.589	11.014	0.58
Pt	−6.382	8.985	0.00
Pa	−1.962	7.613	0.999

**Table 7:** Outcomes of CS-ARDL.

Variables	Long-Run Results		Short-Run Results	
	Coefficients	Standard Error	Coefficients	Standard Error
lnEC	0.0019 ***	0.0014	0.00015 ***	0.00041
lnGDP	0.0018	0.0023	0.00062 *	0.00039
lnHEX	0.0025 ***	0.0008	0.00025 ***	0.00034
lnPOP	−0.2042	0.1423	−0.0572	0.0325
lnCO_2_	−0.0028 ***	0.0021	−0.00088 ***	0.00119
ECT			−0.5689	0.1423

Asterisk signs (* and ***) are employed to denote significance level (10% and 10%).

**Table 8:** Robustness of long-run results.

Variables	AMG	CCEMG
lnEC	0.0181 *** (0.00169)	0.0135 *** (0.00115)
lnGDP	0.0068 (0.00111)	0.0094 (0.00112)
lnHEX	0.0619 *** (0.00143)	0.0864 *** (0.0082)
lnPOP	0.0608 * (0.0425)	−0.00403 (0.0416)
lnCO_2_	−0.0045 *** (0.000958)	−0.00195 *** (0.000614)
Constant	3.371 *** (0.710)	0.690 ** (1.072)
Observations	1104	1104
Number of IDs	46	46

Standard errors in parentheses; Asterisk signs (*, **, and ***) are employed to denote significance level (10%, 5%, and 10%).

## Data Availability

The data will be available on request.

## Abbreviations

ADB	Asian Development Bank
AMG	Augmented mean group
CCEMG	Common correlated effects mean group
CIPS	Conditional inference procedures for stratified samples
CO_2_	Carbon dioxide
CS-ARDL	Cross-sectional autoregressive distributed lag
CSD	Cross-sectional dependence
EG	Economic growth
GDP	Gross domestic product
GHG	Greenhouse gas
GMM	Generalized method of moments
HO	Health outcomes
IRENA	International renewable energy agency
NO_2_	Nitrogen dioxide
OECD	Organization for Economic Cooperation and Development
OLS	Ordinary least squares
PPP	Purchasing power parity
PYLL	Potential years of life lost
REC	Renewable energy consumption
SH	Slope homogeneity
SSA	Sub-Saharan Africa
TB	Tuberculosis
USAID	United States Agency for International Development
WHO	World Health Organization

## Appendix A. List of Asian Countries

Afghanistan, Armenia, Bangladesh, Azerbaijan, Cambodia, Malaysia, Maldives, China, Cyprus, Mongolia, Bahrain, Myanmar, Georgia, Bahrain, Bhutan, India, Saudi Arabia, Nepal, Indonesia, Iran, Brunei Darussalam, Oman, Kazakhstan, Pakistan, Iraq, Philippines, Israel, Korea Republic, Japan, Singapore, Qatar, Jordan, Lebanon, Kuwait, Kyrgyz Republic, Vietnam, Yemen, Uzbekistan, UAE, Turkmenistan, Turkey, Timor-Leste, Thailand, Tajikistan, Syria, and Sri Lanka.
